# Noninvasive fungal rhinosinusitis: a case series

**DOI:** 10.12688/f1000research.67204.2

**Published:** 2022-09-14

**Authors:** Mohamed Masmoudi, Jihene Chelli, Asma Ben Mabrouk, Ezer Chebil, Wadii Thabet, Mehdi Hasnaoui, Khalifa Mighri

**Affiliations:** 1 Department of Otolaryngology - Head and Neck Surgery, Faculty of Medicine of Monastir, Tahar Sfar Hospital, Mahdia, 5100, Tunisia; 2 Department of Endocrinology and Internal Medicine, Faculty of Medicine of Monastir, Tahar Sfar Hospital, Mahdia, 5100, Tunisia

**Keywords:** allergic fungal sinusitis, fungal ball, diagnosis, surgery, treatment

## Abstract

**Introduction: **Fungal rhinosinusitis (FRS) remains a rare disease. The noninvasive forms are hard to diagnose. The management protocols remain controversial. We aim to describe the clinical, radiological and pathological features of noninvasive FRS and present our management protocol and follow-up results.

**Patients and methods: **This descriptive study was conducted in the ear-nose-throat department of the university hospital, Taher Sfar in Mahdia, Tunisia. All patients who responded to the definition of noninvasive FRS (fungal balls and allergic fungal sinusitis) were included. The study was conducted over a three year period (May 2017 – April 2021).

**Results: **Eleven patients were included in this study: four cases of fungal balls and seven cases of allergic fungal sinusitis. Patients presented with symptoms of chronic recurrent rhinosinusitis with no response to conventional treatments. Computed tomodensitometry scan showed opacification of the paranasal sinuses in all patients. Other signs were heterogeneous opacities, local calcifications and thinning of the bony walls of the sinuses.

Histopathological findings were inflammatory polyps in all cases of allergic FRS with the presence of fungal hyphae in 42.8% of the cases.

All patients underwent surgery after a median delay of 12 [6–24] months of the symptom’s onset. The used procedures were endoscopic middle meatal antrostomy for all patients, ethmoidectomy (81.8%) and sphenoidotomy (36.4%). None received systemic antifungals or corticosteroids with a favorable outcome in all cases.

**Conclusion: **Symptoms of noninvasive FRS are nonspecific. The scan images contribute to the diagnosis, but the perioperative findings and the histopathological results remain crucial.  The management is mainly surgical.

## Introduction

Fungal rhinosinusitis (FRS) consists of a group of heterogeneous affections ^1^. At one time FRS was considered a rare disease but its incidence seems to be increasing all over the world ^2^. The diagnosis is particularly difficult. On one hand, the clinical presentation is non-specific and sometimes misleading. On the other hand, fungal colonization of the sinuses is very common making it hard to be certain of its implication in the genesis of the pathology ^3, 4^. The most used classification is based on histopathological evidence of tissue invasion by fungi. It divides FRS into invasive and noninvasive forms. The invasive FRS includes acute invasive, granulomatous invasive FRS, and chronic invasive FRS. The noninvasive FRS includes fungal colonization, fungal ball, and allergic fungal sinusitis ^5^. The aim of this study is to describe the clinical, radiological and pathological features of noninvasive FRS in our ear-nose-throat (ENT) department and present our management protocol and follow-up results.

## Patients and methods

This descriptive study was conducted in the ENT department of the university hospital, Taher Sfar in Mahdia. All patients who responded to the definition of noninvasive fungal rhinosinusitis (fungal balls and allergic fungal sinusitis) according to the 2009 consensus were included ^5^. The study was conducted over a three-year period between May 2017 and May 2021.

The patients’ medical records were consulted and data including demographic characteristics, clinical examination findings, computed tomodensitometry scan results, cytopathology findings and management details were collected.

Computed tomodensitometry scan images were obtained using a General Electric BrightSpeed Elite 16 slice CT scanner. Coronal and sagittal views were reformatted with a 1.25 mm slice thickness.

Magnetic resonance imaging was not available in our hospital, so, it was only realized for patients who could afford to pay for it in the private sector.

Statistical analysis was carried out with Statistical Package for Social Sciences ( SPSS) Statistics, version 23, (IBM Corp., Armonk, NY. Released 2015). The results were expressed as numerical values (percentages) for categorical variables, medians [interquartile range] for continuous variables when Kolmogorov-Smirnov p-value was inferior to 0.05 and means ± standard deviation when Kolmogorov-Smirnov p-value was superior to 0.05. Univariate analysis was performed using the chi-square tests. Co-variates retained in the final model were the ones significant at the level of 5%.

## Results

Eleven patients were included in this study, four cases of fungal balls and seven cases of allergic fungal sinusitis ( Table 1). The median age was 33 [25–42] years with a sex ratio of 0.83. Two patients were diabetics and one patient had asthma.

**Table 1.** Report of the 11 cases of noninvasive fungal rhinosinusitis.

	Age (years)	Gender	Sinusitis type	Scan results	Lund - Mckay score	MRI of the sinuses	Perioperative findings	Surgical procedure	Cytopathology results
1	23	F	AFRS	Unilateral opacification of the ethmoidal, frontal and maxillary sinuses	6	_	Mucin within the ethmoidal and maxillary sinuses	Endoscopic middle meatal antrostomy + anterior ethmoidectomy	Inflammatory polyps + fungal hyphae
2	42	F	AFRS	Bilateral opacification of all sinuses	24	_	Mucin within the two maxillary sinuses	Endoscopic middle meatal antrostomy + total ethmoidectomy + sphenoidotomy	Inflammatory polyps
3	34	F	AFRS	Unilateral opacification of all sinuses	12	_	Mucin within the ethmoidal and maxillary sinuses	Endoscopic middle meatal antrostomy + total ethmoidectomy	Inflammatory polyps + fungal hyphae + Charcot Leyden crystals
4	33	M	AFRS	Bilateral opacification of all sinuses + thinning of the bony walls of the frontal sinus	24	T1: central hypointensity T2: a signal void	Polyps of the middle meatal + mucin	Endoscopic middle meatal antrostomy + total ethmoidectomy + sphenoidotomy	Inflammatory polyps + aspergillus hyphae
5	35	M	AFRS	Unilateral opacification of the ethmoidal, sphenoidal and maxillary sinuses + thinning of the bony walls of the maxillary sinus	6	_	Mucin within the sinuses	Endoscopic middle meatal antrostomy + total ethmoidectomy + sphenoidotomy + septoplasty	Inflammatory polyps
6	30	F	AFRS	Unilateral opacification of all sinuses + calcifications + thinning of the bony walls of the ethmoidal and sphenoidal sinuses	12	T1: central hypointensity T2: a signal void	Mucin within the sinuses	Endoscopic middle meatal antrostomy + total ethmoidectomy + sphenoidotomy	Inflammatory polyps
7	25	M	AFRS	Unilateral opacification of all sinuses + calcifications + thinning of the bony walls of the frontal and sphenoidal sinuses	12	T1: central hypointensity T2: a signal void	Mucin within the sphenoidal and maxillary sinuses	Endoscopic middle meatal antrostomy + total ethmoidectomy + sphenoidotomy + septoplasty	Inflammatory polyps
8	33	F	FB	Unilateral opacification of all sinuses + calcifications	12	T1: isointensity T2: hyper intensity	Scattered purulent material within the ethmoidal and maxillary sinuses	Endoscopic middle meatal antrostomy + total ethmoidectomy	Inflammatory polyps + aspergilloma
9	62	M	FB	Unilateral opacification of the maxillary sinus + a thickening of the bony walls	2	_	Scattered purulent material within the maxillary sinus	Endoscopic middle meatal antrostomy + septoplasty	Masses of aspergillosis mycelia, inflammatory cells, fibrin and mucus
10	56	M	FB	Unilateral opacification of the maxillary sinus + calcifications + a thickening of the bony walls	2	_	Scattered purulent material within the maxillary sinus	Endoscopic middle meatal antrostomy + turbinoplasty	Masses of aspergillosis mycelia, inflammatory cells, fibrin and mucus
11	25	F	FB	Unilateral opacification of the ethmoidal, frontal and maxillary sinuses + thickening of the bony walls of the sinuses	6	_	Necrotic formations of the ethmoidal and maxillary sinuses	Endoscopic middle meatal antrostomy + anterior ethmoidectomy	Inflammatory polyps

F= Female, M= Male, AFRS= Allergic fungal rhinosinusitis, FB= Fungal ball, MRI= Magnetic resonance imaging

None of the patients reported a history of dental infection or endodontic procedure.

The median delay between the consultation and the symptom’s onset was 12 [9–60] months. The main symptoms, patients presented with, were nasal obstruction (N=9; 81.8%), rhinorrhoea (N=5; 45.5%), anosmia (N=4; 36.4) and epistaxis (N=3; 27.3%).

None of the patients presented with fever. Anterior rhinoscopy was performed for all patients. Polyps were found in six cases (54.5%). The presence of mucopurulent drainage was noted in four patients (36.4%). A septal deviation was found in four cases (36.4%).

**Figure 1.**
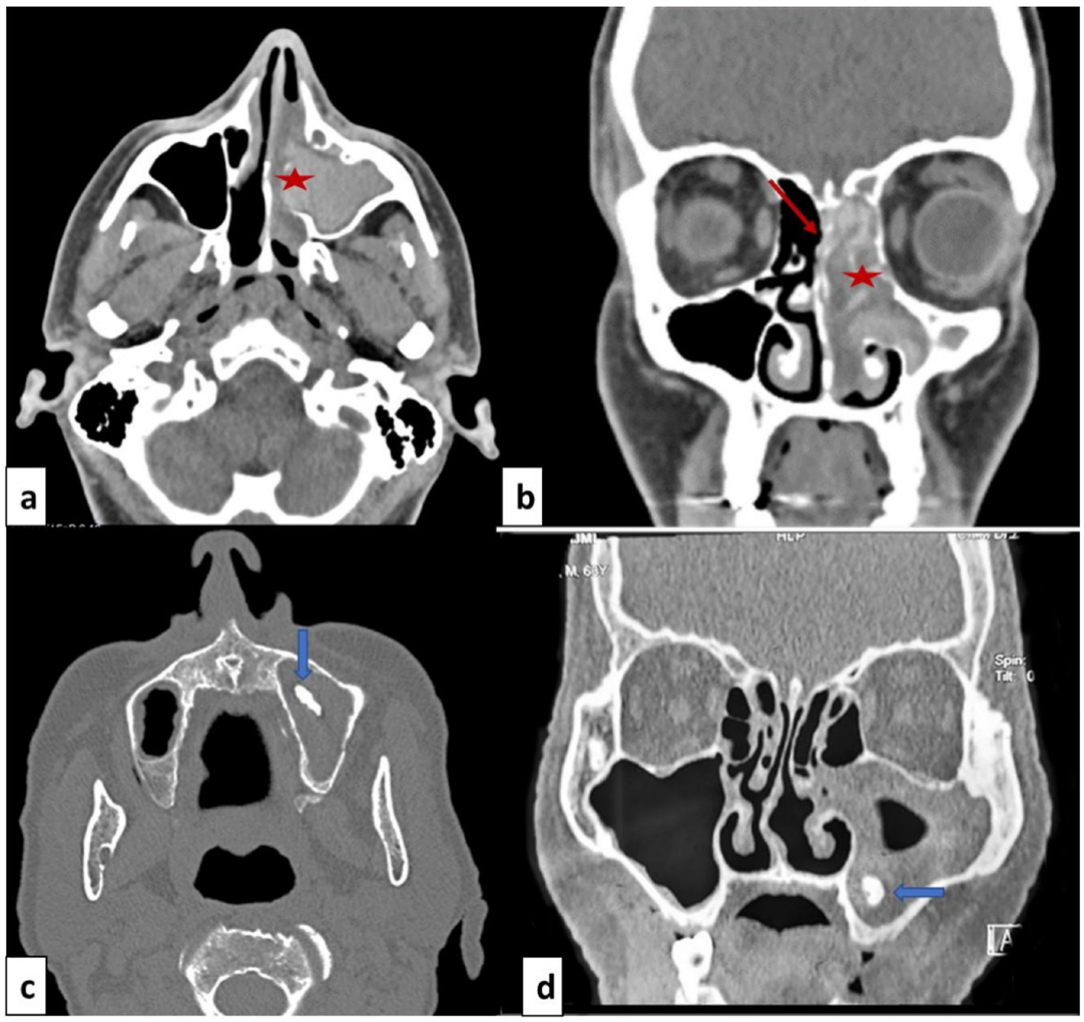
Facial mass computed tomodensitometry with axial cut ( **a**, **c**) and coronal cut ( **b**, **d**). ( **a**, **b**): Allergic fungal rhinosinusitis: heterogeneous opacity of the nasal cavity and ethmoido-maxillary cavities (red star) and thinning of the bony walls (red arrow). ( **b**, **d**): Fungal ball: opacity of the right maxillary sinus with local calcification in maxillary sinus (blue arrow).

Computed tomodensitometry scan showed opacification of the paranasal sinuses in all patients, unilateral in eight cases (72.7%) and bilateral in three cases (27.3%). The affection consisted of pansinusitis in eight cases (72.7%). The other radiological signs were heterogeneous opacities within the sinus cavity (N=5; 45.5%), local calcifications (N=4; 36.4%) and thinning of the bony walls of the sinuses (N=4; 36.4%) ( Figure 1).

**Figure 2.**
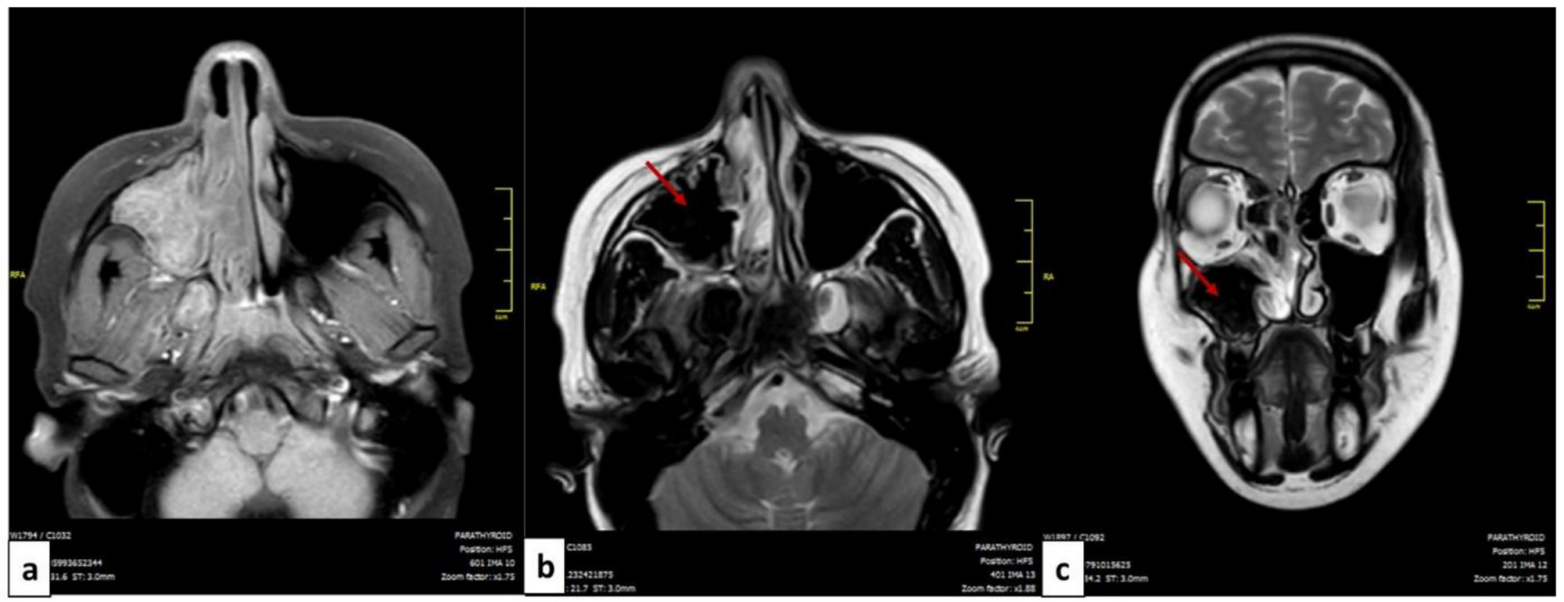
MRI of the facial mass of a patient with allergic fungal rhinosinusitis: axial cut ( **a**, **b**) and coronal cut ( **c**). The filling of the left maxillary sinus is presented in isosignal T1 ( **a**) and in hyposignal T2 or signal void (red arrow) ( **b**, **c**).

MRI of the facial mass was performed in four patients. In all cases, it showed T2 hypointense (signal void) images suggestive of the mycotic origin ( Figure 2).

Histopathological findings were inflammatory polyps in all cases of allergic FRS with the presence of fungal hyphae in 45.8% of the cases. For fungal balls, masses of aspergillosis mycelia along with inflammatory cells were found in 75% of the cases ( Table 1).

**Figure 3.**
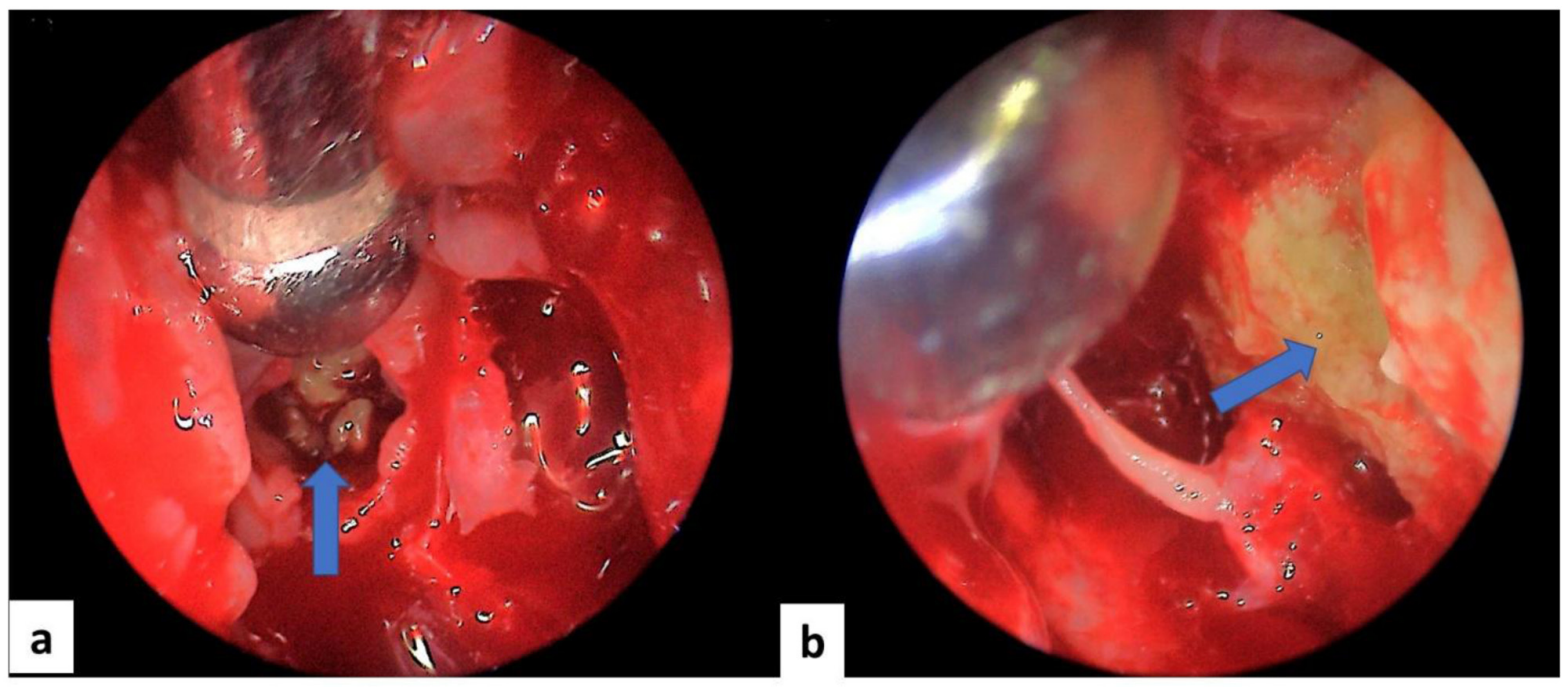
Intraoperative endoscopic view of a case of allergic fungal rhinosinusitis: mucin (blue arrow) within ethmoidal cells ( **a**) and of the left maxillary sinus ( **b**).

**Figure 4.**
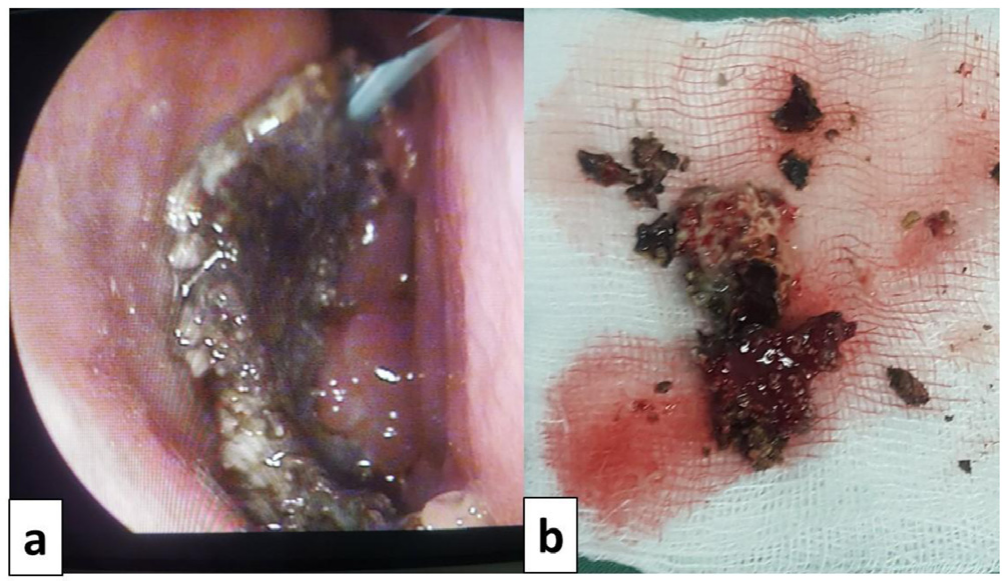
Intraoperative view of a case of fungal ball: **a**: fungal ball taken from the left maxillary sinus. **b**: fungal ball specimen.

All patients underwent surgery after a median delay of 12 [6–24] months of the symptom’s onset. The used procedures were endoscopic middle meatal antrostomy for all patients (100%), ethmoidectomy for nine patients (81.8%), sphenoidotomy for four patients (36,4%), septoplasty for three patients (27.3%) and turbinoplasty for one patient (9.1%). Details of the surgical procedures and perioperative findings are presented in Table 1, Figure 3 and Figure 4. None of the patients received systemic antifungal treatment. All were prescribed a local treatment with nasal saline topical. The seven patients diagnosed with allergic FRS received nasal steroid sprays (63.6%). After a median follow-up delay of three [1–12] months, all patients had a favorable outcome, and no recurrence was reported during this period. However, in the case of allergic FRS, the recovery seemed to take more time, and symptoms seemed to persist a little longer than the fungal ball cases.

None of the symptoms such as nasal obstruction, rhinorrhoea, anosmia and epistaxis, was associated with either of the forms (fungal balls or allergic fungal sinusitis) with respective p-values of 1, 1, 0.2 and 1.

## Discussion

Although sinusitis is very common, fungal sinusitis remains rare, but the prevalence seems to be rising all over the world, *Aspergillus* sp. being the most implicated fungal agent ^6^.

The fungi’s implication in rhinosinusitis has been widely discussed and many diagnostic criteria have been proposed in order to identify the different forms. The most used classification is the one dividing FRS into invasive and noninvasive sinusitis based on histopathological findings. On one hand, the invasive forms include acute invasive FRS, granulomatous invasive FRS, and chronic invasive FRS. On the other hand, noninvasive forms include saprophytic fungal infestation, fungal ball, and fungus related-eosinophilic FRS, mainly allergic FRS ^5^. In this study, we only focused on noninvasive FRS presenting as fungal balls or allergic fungal sinusitis. This pathology, as found in our results, mainly occurs among young adults ^7^.

In line with our findings, individuals with allergic FRS or fungal balls are reported to present with the typical symptoms of chronic rhinosinusitis, including nasal congestion, facial pain when pressuring the sinuses, and nasal discharge ^1^. The symptoms are chronic (> three months), recurrent and resistant to conventional treatments usually prescribed for chronic rhinosinusitis ^6^. These two forms, despite their common clinical presentation, have different physiopathology. In the case of fungal balls, the fungi colonizing the sinus mucosa produce a dense conglomeration of hyphae within the maxillary sinus or, less frequently, in the sphenoid sinus ^8^. In the case of allergic FRS, a profound Th2 lymphocyte response associated with eosinophilic mucin within the sinus was noted ^8^. Many studies suggest that fungi may have an effect on sinus mucosa in susceptible individuals. The absence of convincing immunological data and the controversial role of antifungal agents make it hard to conclude the real role of fungi in the genesis of allergic FRS ^3^. Some suggest that the mechanism is similar to allergic bronchopulmonary aspergillosis, fungal antigens induce a type I hypersensitivity reaction with the production of IgE, IgA and IgG ^6^.

According to the recent classification of Chronic Rhinosinusitis, the fungal ball is most often related to an endodontic history. For the 4 cases of fungal ball in our series, there was no history of dental infection nor procedure ^9^. All patients were referred to a dentist for a specialized dental examination and eventual treatment of a dental cause to reduce the risk of recurrence.

Anterior rhinoscopy findings reported in the literature are congruent with our findings, mainly congestive mucosa, mucopurulent discharge and polyps especially in the case of allergic FRS ^10^.

The computed tomodensitometry scan images contribute to the diagnosis. In the case of fungal balls, the affection mostly concerns the maxillary sinus. The most reported features were calcifications within the maxillary sinus followed by complete opacification, partial opacification with an irregular surface and bony sclerosis or bone thickening ^11^. These signs remain nonspecific and could be seen in sinusitis of other origins or neoplasms. The MRI is more performant, the fungal ball is hypointense on T1-weighted and T2-weighted images. In the case of allergic FR, scan images typically show bilateral pansinusitis. The opacification of the sinuses is explained by the hyperattenuated mucin. In many cases, as noted for our patients, expansion and thinning of sinus walls were reported. T1-weighted MRI images may show mixed signal intensities. T2-weighted images are mostly hypointense but may show flow voids ^1^.

Histopathological findings are the main diagnostic criterion. It is the only element to confirm the noninvasive form of the disease. The other findings reported were copious mucin, abundant eosinophils, Charcot-Leyden crystals, with rare noninvasive fungal hyphae in case of allergic FRS. Tightly packed fungal hyphae without allergic mucin are the characteristic feature of fungal balls ^12^.

The results of fungal cultures were not presented in our study. In the literature, *Aspergillus fumigatus* is the most incriminated fungal species in the genesis of fungal balls, but the fungal cultures were negative in 65% of cases. In allergic FRS, the use of type I hypersensitivity testing is a fundamental diagnosis criterion. This test is unfortunately unavailable in our settings. Fungal cultures are not useful since studies have shown a difference between the fungal species cultured and fungal-specific sensitivities revealed by the allergy testing ^1^.

The diagnosis of fungal balls is easily confirmed when there is radiological evidence of sinus opacification with or without radiographic heterogeneity, a mucopurulent or necrotic material in the sinus and a histological aspect associating a dense conglomeration of hyphae and inflammation of the mucosa without invasion ^5^.

The diagnosis of allergic FRS is less evident, based on the association of many criteria; type I hypersensitivity to fungi, nasal polyposis, fungi on staining, eosinophilic mucin without fungal invasion into sinus tissue, and characteristic radiological findings on CT scanning ^6^. The determination of serum IgE level could be an additional element in the diagnosis ^13^. But, this biomarker was not available in our laboratory.

The management of noninvasive FRS is mainly surgical. Surgical opening of the natural sinus ostium with the evacuation of fungal debris is the treatment of choice in the case of fungal balls. An inferior meatal window combined with the middle meatal antrostomy could reduce the risk of leaving residual debris ^13^. After the removal of fungal hyphae, no additional treatment is necessary according to most recommendations ^1, 6^.

The management of allergic FRS is less consensual. Surgical management was adopted by most series to remove polyps, open sinus ostia, and clear the eosinophilic fungal mucin.

Many medical therapies were tried in the management of allergic FRS. Oral corticosteroid therapy over a period of 2 to 6 months, with close monitoring of side effects and topical steroids were the most used medications with a reported improvement of the symptoms and reduction of recurrences ^13^. Oral antifungals such as voriconazole and itraconazole and topical antifungal agents have been proposed by some authors, especially, in immunocompromised patients in order to prevent invasive forms of FRS. However, these therapies remain unsupported by solid data ^6, 14^. Leukotriene antagonists and immunotherapy have also been used with no evidence of a better outcome ^1, 15^. Our management protocol was based on endoscopic surgery with postoperative topical steroids with a favorable outcome.

This study is, to our knowledge, the first to report cases of noninvasive FRS in Tunisia. This disease, once rare, is reported more and more. Our management protocol seems to have good results, but we conducted a single-center study with a limited number of cases. On the other hand, the follow-up period was limited, and recurrences may occur in the future. Multicenter studies with a longer follow-up could refine the results and help draw better conclusions.

## Conclusion

Noninvasive FRS is to be evoked when a patient presents with symptoms of chronic recurrent rhinosinusitis with no response to conventional treatments. Scan images could help steer toward the diagnosis, but the perioperative findings and the histopathological results remain crucial. Surgical treatment is the milestone in the management of FRS.

## Consent

Written informed consent for publication of their clinical details and clinical images was obtained from the patients.

## Data Availability

All data underlying the results are available as part of the article and no additional source data are required.
