# Supplementary material for: Noninvasive fungal rhinosinusitis: a case series
Source: F1000Res. 2022 Sep 14;10:869. Originally published 2021 Aug 31. [Version 2] doi: 10.12688/f1000research.67204.2 (PMC9525990; doi:10.12688/f1000research.67204.2)
Supplement: Peer review history

# Peer review: Reviewer response for version 2

**DOI:** 10.5256/f1000research.136141.r150422

**Published:** 2022-09-30

**Authors:** Yasser M. Elbeltagy

**Affiliations:** 1 Department of Otorhinolaryngology, Ain Shams University, Cairo, Egypt

The authors mentioned reasonable explanations to my questions. I accept their answers. Thank you for your reply.

Are enough details provided of any physical examination and diagnostic tests, treatment given and outcomes?

No

Is the background of the cases’ history and progression described in sufficient detail?

Partly

Is sufficient discussion included of the importance of the findings and their relevance to future understanding of disease processes, diagnosis or treatment?

Partly

Is the conclusion balanced and justified on the basis of the findings?

Partly

Reviewer Expertise:

Rhinology

I confirm that I have read this submission and believe that I have an appropriate level of expertise to confirm that it is of an acceptable scientific standard.

# Peer review: Reviewer response for version 1

**DOI:** 10.5256/f1000research.70803.r93107

**Published:** 2021-11-22

**Authors:** Yasser M. Elbeltagy

**Affiliations:** 1 Department of Otorhinolaryngology, Ain Shams University, Cairo, Egypt

1. The nasal endoscopic examination should be mentioned & performed in the study design, not only anterior rhinoscopy.
2. Concerning imaging studies used in the article, the authors did not mention MRI in the study design. They mentioned it later in the results that made the matter perplexing.
3. Concerning the evaluation of CT findings in cases studied, I would prefer Lund - Mckay scores for the accurate evaluation of the severity of sinusitis numerically rather than a descriptive one.
4. The authors did not mention the progressive course of illness of the 2 patients (the diabetic one & the asthmatic one)  as they are expected to have severer presentation than other ones.

Are enough details provided of any physical examination and diagnostic tests, treatment given and outcomes?

No

Is the background of the cases’ history and progression described in sufficient detail?

Partly

Is sufficient discussion included of the importance of the findings and their relevance to future understanding of disease processes, diagnosis or treatment?

Partly

Is the conclusion balanced and justified on the basis of the findings?

Partly

Reviewer Expertise:

Rhinology

I confirm that I have read this submission and believe that I have an appropriate level of expertise to confirm that it is of an acceptable scientific standard, however I have significant reservations, as outlined above.

# Response

**Published:** 2022-07-12

**Authors:** Mohamed Masmoudi

**Affiliations:** Faculty of Medicine of Monastir, Tunisia

The nasal endoscopic examination should be mentioned & performed in the study design,

 not only anterior rhinoscopy.

**Author response**: Anterior rhinoscopy and nasal endoscopic examination were performed for all patients. It was an omission in the initial manuscript.

 Concerning imaging studies used in the article, the authors did not mention MRI in the

 study design. They mentioned it later in the results that made the matter perplexing.

 ​​​​​​​

**Author response**: Magnetic resonance imaging was not available in our hospital, so, it was only realized for patients who could afford to pay for it in the private sector. We added this sentence to the manuscript. 

 Concerning the evaluation of CT findings in cases studied, I would prefer Lund - Mckay

 scores for the accurate evaluation of the severity of sinusitis numerically rather than a

 descriptive one.

 ​​​​​​​

**Author response**: The score was calculated and added to table I.

 The authors did not mention the progressive course of illness of the 2 patients (the diabetic

 ones (3 + 10) & the asthmatic one (2) as they are expected to have a severer presentation than other ones.

 ​​​​​​​

**Author response**: The two diabetic patients (3 and 10 in the table ) and the asthmatic one (2 in the table) did not have anything in particular concerning their presentation or their outcome.

 Thank you for your advice that helped improve this manuscript.

# Peer review: Reviewer response for version 1

**DOI:** 10.5256/f1000research.70803.r93106

**Published:** 2021-09-17

**Authors:** Senda Turki

**Affiliations:** 1 Service ORL, Hôpital FSI,Faculté de Médecine de Tunis, Université Tunis El Manar, Tunis, Tunisia

The topic addressed in this study is particularly interesting because it deals with a frequent pathology whose diagnosis is difficult and whose early treatment makes it possible to avoid the evolution to serious complications. If the treatment is undoubtedly based on a complete surgical procedure, the place of an associated medical treatment (whether preoperative or postoperative) remains a matter of controversy.

 This study is easy to follow, well-structured, and illustrated with endoscopic views and well-captioned radiologic images. Given the fact that it is a small series, the statistical study is considered to be modest. On the other hand, the different cases are very clearly presented in Table 1. The main points are correctly detailed in the discussion section.

 However, I have a few remarks that the authors might consider:

1. According to the recent classification of Chronic Rhinosinusitis (CRS), the fungal ball is a secondary CRS and is most often related to an endodontic history (more than 80% of cases). For the 4 fungal balls of the series, there is no information regarding the dental history, the specialized dental examination, the dentascan. The treatment of dental causes should be combined with surgical treatment to reduce the risk of recurrence. “Contemporary Classification of Chronic Rhinosinusitis Beyond Polyps vs No Polyps: A Review” ^1^. Jessica W Grayson, Claire Hopkins, Eri Mori, Brent Senior, Richard J Harvey.
2. An inferior meatal window combined with the middle meatal antrostomy for the treatment of fungal balls would reduce the risk of leaving residual debris (EPOS2020) ^2^
3. An MRI was requested in 4 patients: What were the clinical or radiologic criteria that motivated the request for an additional MRI? What were the radiological elements that the MRI had to specify?
4. Concerning the cases of allergic fungal rhinosinusitis: a/ The determination of serum IgE level would be an additional element in the diagnosis. b/ Postoperative oral corticosteroid therapy over a period of 2 to 6 months (with close monitoring of side effects) demonstrated a net benefit on faster amendment of symptoms and reducing recurrences (EPOS2020) ^2^
5. You could have added in the references, The European Position Paper on Rhinosinusitis and Nasal Polyps 2020 ^2^ (the update of similar evidence-based position papers published in 2005 and 2007 and 2012): EPOS2020.

By no means do the remarks diminish the value of this work which, as the authors propose, should be complemented by a multicenter Tunisian study with greater follow-up in order to optimize diagnostic and therapeutic strategies.

Are enough details provided of any physical examination and diagnostic tests, treatment given and outcomes?

Yes

Is the background of the cases’ history and progression described in sufficient detail?

Yes

Is sufficient discussion included of the importance of the findings and their relevance to future understanding of disease processes, diagnosis or treatment?

Yes

Is the conclusion balanced and justified on the basis of the findings?

Yes

Reviewer Expertise:

Rhinology, Otology, Balance diseases and vertigo, Sleep Apnea, Neck surgery

I confirm that I have read this submission and believe that I have an appropriate level of expertise to confirm that it is of an acceptable scientific standard.

## References

[1] : Contemporary Classification of Chronic Rhinosinusitis Beyond Polyps vs No Polyps. *JAMA Otolaryngology–Head & Neck Surgery* .2020;146(9) : 10.1001/jamaoto.2020.1453 10.1001/jamaoto.2020.1453 32644117

[2] : European Position Paper on Rhinosinusitis and Nasal Polyps 2020. *Rhinology* .2020;58(Suppl S29) : 10.4193/Rhin20.600 1-464 10.4193/Rhin20.600 32077450

# Response

**Published:** 2022-07-12

**Authors:** Mohamed Masmoudi

**Affiliations:** Faculty of Medicine of Monastir, Tunisia

According to the recent classification of Chronic Rhinosinusitis (CRS), the fungal ball is a

 secondary CRS and is most often related to an endodontic history (more than 80% of cases).

 For the 4 fungal balls of the series, there is no information regarding the dental history, the

 specialized dental examination, the dentascan. The treatment of dental causes should be

 combined with surgical treatment to reduce the risk of recurrence. “Contemporary

 Classification of Chronic Rhinosinusitis Beyond Polyps vs No Polyps: A Review”1. Jessica W

 Grayson, Claire Hopkins, Eri Mori, Brent Senior, Richard J Harvey.

**Author response**: We specified that none of our patients had a history of dental infection or procedure. We added in the discussion part that they were all referred to a dentist for a specialized examination.

 An inferior meatal window combined with the middle meatal antrostomy for the treatment

 of fungal balls would reduce the risk of leaving residual debris (EPOS2020)2

**Author response**: This was added to the discussion. 

 An MRI was requested in 4 patients: What were the clinical or radiologic criteria that

 motivated the request for an additional MRI? What were the radiological elements that the

 MRI had to specify?

 ​​​​​​​

**Author response**: We added in the method part the limitation we had since MRI is not available in our center. So it was only indicated for patients who could afford it for further specifications.

 Concerning the cases of allergic fungal rhinosinusitis: a/ The determination of serum IgE

 level would be an additional element in the diagnosis. b/ Postoperative oral corticosteroid

 therapy over a period of 2 to 6 months (with close monitoring of side effects) demonstrated

 a net benefit on faster amendment of symptoms and reducing recurrences (EPOS2020)2

 ​​​​​​​

**Author response**: This information was added, the serum IgE level was not possible, since unavailable. 

 You could have added in the references, The European Position Paper on Rhinosinusitis and

 Nasal Polyps 20202 (the update of similar evidence-based position papers published in 2005

 and 2007 and 2012): EPOS2020.

 ​​​​​​​

**Author response**: These references were added.

 Thank you for your remarks that showed the special interest you paid to our manuscript and helped improve its quality.
